# Gastric insufflation and surgical view according to mask ventilation method for laparoscopic cholecystectomy: a randomized controlled study

**DOI:** 10.1186/s12871-023-02269-9

**Published:** 2023-09-20

**Authors:** Yun Kyung Jung, Cho Long Kim, Mi Ae Jeong, Jeong Min Sung, Kyeong Geun Lee, Na Yeon Kim, Leekyeong Kang, Hyunyoung Lim

**Affiliations:** 1 Department of Surgery, Hanyang University Hospital, Hanyang University College of Medicine, Seoul, Republic of Korea; 2 Department of Anesthesiology and Pain Medicine, Hanyang University Hospital, Hanyang University College of Medicine, 222-1, Wangsimni-ro, Seoungdong-gu, 04763 Seoul, Republic of Korea

**Keywords:** Anesthesia induction, Gastric antral cross-sectional area, Gastric insufflation, Gastric ultrasound, Laparoscopy, Rapid sequence induction

## Abstract

**Background:**

Proper mask ventilation is important to prevent air inflow into the stomach during induction of general anesthesia, and it is difficult to send airflow only through the trachea without gastric inflation. Changes in gastric insufflation according to mask ventilation during anesthesia induction were compared.

**Methods:**

In this prospective, randomized, single-blind study, 230 patients were analyzed to a facemask-ventilated group (Ventilation group) or no-ventilation group (Apnea group) during anesthesia induction. After loss of consciousness, pressure-controlled ventilation at an inspiratory pressure of 15 cmH2O was performed for two minutes with a two-handed mask-hold technique for Ventilation group. For Apnea group, only the facemask was fitted to the face for one minute with no ventilation. Next, endotracheal intubation was performed. The gastric cross-sectional area (CSA, cm^2^) was measured using ultrasound before and after induction. After pneumoperitoneum with carbon dioxide, gastric insufflation of the surgical view was graded by the surgeon for each group.

**Results:**

Increase of postinduction antral CSA on ultrasound were not significantly different between Ventilation group and Apnea group (0.04 ± 0.3 and 0.02 ± 0.28, p-value = 0.225). Additionally, there were no significant differences between the two groups in surgical grade according to surgeon’s judgement.

**Conclusions:**

Pressure-controlled ventilation at an inspiratory pressure of 15 cmH_2_O for two minutes did not increase gastric antral CSA and insufflation of stomach by laparoscopic view.

**Trial Registration:**

http://cris.nih.go.kr (KCT0003620) on 13/3/2019.

## Introduction

Mask ventilation before tracheal intubation has been considered essential to prevent hypoxia in patients [1, 2]. For patients with the risk factors of pulmonary aspiration, such as full stomach or laryngeal incompetence, the technique of rapid sequence induction by compressing the cricoid cartilage to close the esophagus and open the airway only for mask ventilation or by no mask ventilation for a short time is recommended [2, 3]. If mask ventilation is performed excessively, air enters the stomach as well as the trachea, increasing the risk of hypoxia and pulmonary aspiration due to reflux of gastric contents. However, even with proper mask ventilation in normal patients with sufficient fasting time, pulmonary aspiration can occur because the amount of airflow into the airway is different due to various reasons such as the anatomical diversity and the slow gastric emptying time [1, 4]. Therefore, proper mask ventilation is a challenge for clinicians performing tracheal intubation.

During laparoscopic surgery, if the stomach is inflated excessively due to inadequate mask ventilation, it obstructs the surgeon’s view of the operation, making the operation more difficult and longer. In severe cases, artificial decompression through a Levin tube is required. Therefore, proper mask ventilation is necessary to prevent air inflow into the stomach and improve the operating view.

The gastric antral cross-sectional area (CSA) using gastric ultrasound can be measured more easily than other locations (body or fundus) in the stomach and is closely correlated with gastric content volume [5–8]. Recent studies have reported that a quantitative approach by measuring the antral area and a qualitative examination of the antrum enable a reliable estimate of gastric contents during the perioperative period [1, 9, 10]. Additionally, gastric ultrasound is noninvasive, easy to perform at the bedside with fewer limitations, and helpful to avoid radiation exposure. Therefore, gastric ultrasound is a useful tool for assessing the risk of pulmonary aspiration during the perioperative period.

There have been studies confirming gastric contents through gastric ultrasound [1, 11, 12], but studies confirming air inflow into the stomach are rare, and there is no study directly confirming the degree of gastric distension through laparoscopy. Therefore, we hypothesized that postinduction gastric CSA increases during mask ventilation compared to no ventilation. In addition, we checked the insufflation of stomach by laparoscopic view.

## Methods

This prospective, randomized, single-blind, parallel-groups study was approved by the Institutional Review Board and written informed consent was obtained from all subjects participating in the study. The study was registered prior to patient enrollment at http://cris.nih.go.kr. All procedures were conducted in accordance with the Helsinki Declaration-2013. This manuscript adheres to the CONSORT 2010 statement [13]. The study was conducted from August 2019 to December 2020. A total of 293 patients undergoing laparoscopic cholecystectomy were assessed for eligibility, and 252 patients were enrolled by study staff. Eligible patients were 20–75 years old and undergoing laparoscopic cholecystectomy with a body-mass index less than 30 kg/m2. The exclusion criteria were American Society of Anesthesiologists (ASA) physical status of at least IV, having risk of hypoxia or gastroesophageal reflux (expected or history of difficult intubation, < 92% oxygen saturation before induction, fasting time < 8 h, pregnancy, history of gastroesophageal surgery, and loss of consciousness), and enrollment refusal. Drop-out criteria were change from laparoscopic surgery to open surgery, unclear gastric antrum findings on ultrasound, and failure to insert a Levin tube.

### Randomization and blinding

Participants were randomly allocated to one of two groups (Ventilation group and Apnea group) in a 1:1 ratio. Randomization was done by computer-generated random numbers with a fixed block size of eight and a 1:1 ratio. Randomized numbers were sealed in an opaque envelope. The investigator who was not involved in randomization and allocation opened the sealed envelope immediately before the study and performed anesthesia induction. Other investigators who were blinded to group allocation assessed the gastric antral CSA using ultrasound. They left the operating room and remained blinded during anesthesia induction. Other anesthesiologists who were not involved in the study recorded patient vital signs and ventilator parameters.

### Study protocol and anesthesia

All participants were premedicated with intramuscular atropine (0.01 mg/kg) and midazolam (0.05 mg/kg) one hour before anesthetic induction. After entering the operating room, all participants were monitored for blood pressure, heart rate, oxygen saturation, and anesthesia depth. The level of consciousness and the depth of anesthesia were monitored using a patient state index (PSi) monitor (SedLine® Brain Function Monitoring, Version 1.8.1.4i, Masimo Inc., Irvine, CA, USA). The participants breathed 100% oxygen for three minutes and then started anesthesia induction when end-expiratory oxygen concentration was more than 90%. For anesthesia induction, a 1% lidocaine (30 mg) and propofol (1.0–1.5 mg/kg) bolus with continuous remifentanil infusion (0.05 mg/kg/minute) was used. In the Ventilation group, after loss of consciousness, participants received rocuronium (0.6 mg/kg) intravenously. The junior anesthesiologist performed two-handed mask ventilation 15 times/min in pressure-controlled mode (15 cmH2O) for two minutes with 100% oxygen and inhalational anesthesia using 2.0 vol% of sevoflurane. In the Apnea group, rocuronium (1.0 mg/kg) was administered after loss of consciousness, and 100% oxygen was maintained at 6 L/min through a facemask without positive pressure ventilation, while maintaining apnea for one minute before performing endotracheal intubation. The senior anesthesiologists measured gastric antral CSA using ultrasound. An 18-Fr Levin tube was then inserted by the same anesthesiologist who performed anesthesia induction. The Levin tube was inserted at least 60 cm and its position was confirmed through video laryngoscope that the Levin tube was accurately positioned in the esophagus.

The surgeon evaluated the degree of gastric dilatation after carbon dioxide injection for laparoscopic surgery. Afterward, the anesthesiologist measured the amount of suctioned gastric contents and air using a 50-mL syringe through the Levin tube.

### Ultrasonographic measurements

We manually measured the perimeter (cm) of the cross-section area of the gastric antrum using ultrasonography (SonoSite, Inc., Bothwell, WA, USA) with a low frequency (2–5 MHz) convex transducer in the abdominal view mode, and we automatically calculated the CSA (cm^2^) from ultrasonography. All patients were lying in a supine position on the operating table. The ultrasound assessors were two senior anesthesiologists (MAJ and HL) who had each performed over 100 gastric ultrasound examinations.

**Fig. 1:**
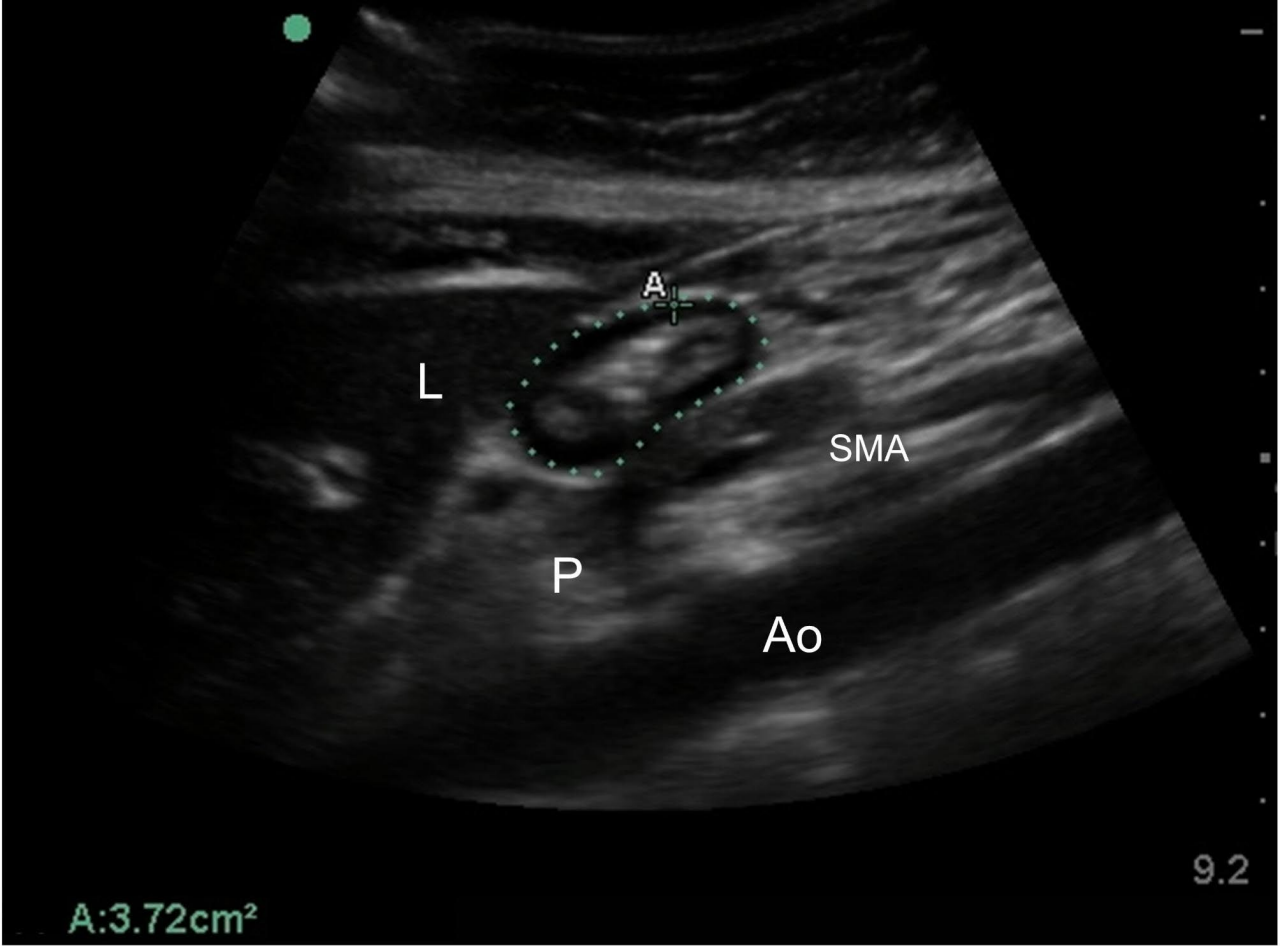
Example of gastral antrum images on ultrasonography. A, antrum of stomach; L, left lobe of liver; Ao, descending aorta; SMA, superior mesenteric artery; P, pancreas

The perimeter (the outermost serosa layer border) of a single section of the gastric antrum was imaged in a parasagittal plane in the epigastric area using the left lobe of the liver, the descending aorta, and the superior mesenteric artery as internal landmarks (Fig. 1). In this view, the assessors measured the CSA using the free-hand tracing tool of the SonoSite ultrasound machine.

### Other outcome measurements

**Fig. 2:**
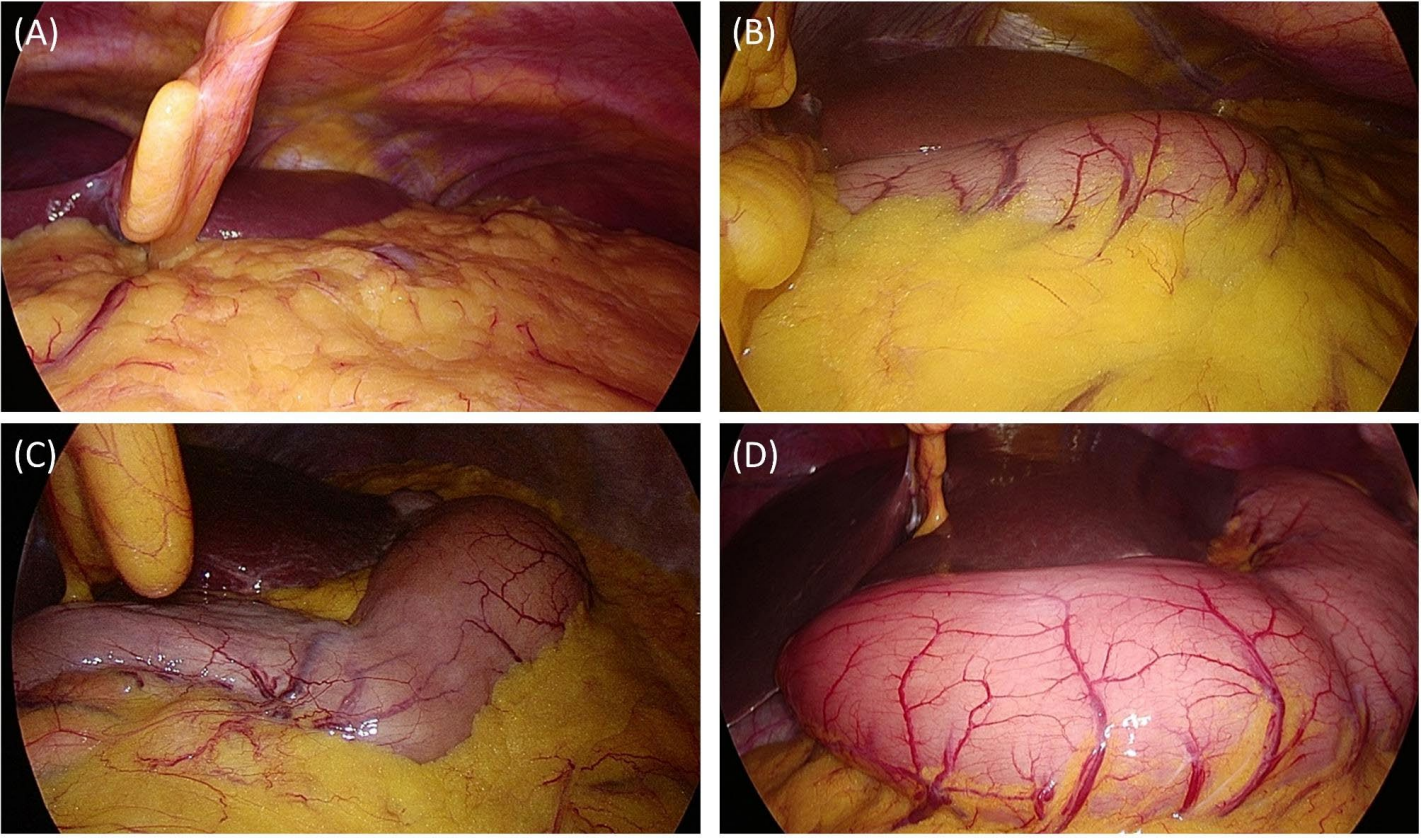
Laparoscopic surgical grade of gastric insufflation. **(A)** Grade 1 = little gastric insufflation. **(B)** Grade 2 = stomach is expanded beyond the lower left end of the liver, but does not go beyond the round ligament. **(C)** Grade 3 = stomach is expanded beyond the lower left end of the liver and goes beyond the round ligament. **(D)** Grade 4 = stomach expands and goes beyond the round ligament, and it is impossible to secure the view around the cystic duct

After pneumoperitoneum using carbon dioxide (CO_2_), an operator who was blinded to the groups recorded gastric insufflation degree. There is no standardized method for surgical view yet. The criteria for classifying the grade were determined based on the experience and knowledge of the surgeon who performed laparoscopic cholecystectomy for more than 30 years. All evaluations were performed at the intraperitoneal pressure of 12 mmHg in the supine position with the same laparoscopy (OTV-S300 & UHI-3, Olympus corporation, Tokyo, Japan). Gastric insufflation degree was classified as follows: Grade 1 = little gastric insufflation, and the stomach visible only under the left lower end of the liver. Grade 2 = slight insufflation, but surgery not affected. Stomach expanded beyond the lower left end of the liver, but does not go beyond the round ligament. Grade 3 = degree to which surgery is possible without manipulation such as artificial decompression, although it causes discomfort in surgery due to gastric insufflation. Stomach expanded beyond the lower left end of the liver and beyond the round ligament. Grade 4 = degree to which surgery is impossible without manipulation (e.g., artificial decompression). Stomach expanded beyond the round ligament, and impossible to secure the view around the cystic duct (Fig. 2).

The amounts of gastric contents and gas through Levin tube suction was also measured after surgical grading. A 50 cc syringe was connected to Levin tube and pulled to aspirate the gastric contents and gas. Additionally, vital signs including oxygen saturation and PSi were checked at five time points from before to after induction: (1) immediately after entering operating room; (2) before induction; (3) immediately before intubation; (4) immediately after intubation; and (5) one minute after intubation.

### Statistical analysis

The sample size was calculated using PASS software (NCSS, version 2021). In a previous study [14], gastric antral CSA before anesthesia induction was 4.02 ± 2.76 cm2 for adult patients in a supine position. We expected a 30% increase in gastric insufflation in the ventilation group than in the apnea group. One hundred eleven patients in each group were needed for 90% power, and the type I error was set at 0.05. Predicting a dropout rate of 10%, 248 patients were deemed sufficient to detect significant differences between groups. Categorical variables are expressed as numbers and percentages. Continuous variables are reported as means ± standard deviations. Normally distributed data were evaluated with the Shapiro-Wilk test or the Kolmogorov-Smirnov test.

Gastric antral area changes on ultrasonography and Levin tube suction amount were evaluated with the Mann-Whitney U test, and surgical grade determined by the surgeon’s judgement was evaluated using the chi-square test.

Demographic data, perioperative data, and clinical outcomes between the two groups were examined with the chi-square test for categorical variables and an independent-samples t-test or Mann-Whitney U test for continuous variables. Vital sign changes during the study were analyzed by repeated measures general linear model, and Spearman correlation analysis was performed to evaluate gastric antral CSA changes on ultrasonography, surgeon’s view grade, and Levin tube suction amount. Data were analyzed using SPSS (Version 24; IBM, Chicago, IL, USA). Two-sided alpha of 0.05 was used for all statistical tests.

## Results

**Fig. 3:**
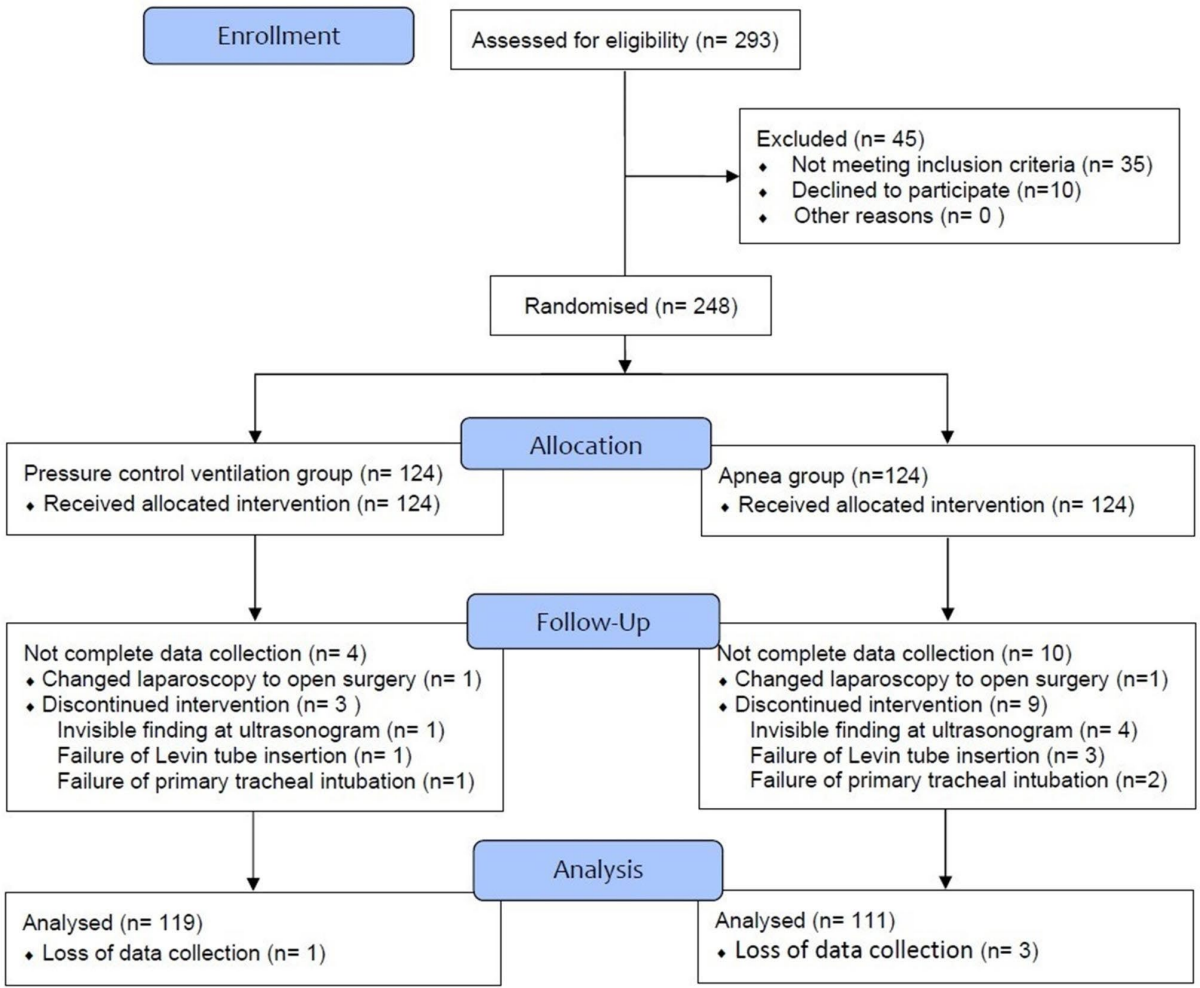
Flow diagram of patient selection in this study

Two hundred ninety-three patients were assessed for eligibility, and 248 patients were enrolled in the study. Five and thirteen patients in the Ventilation and Apnea groups, respectively, dropped out due to changes in surgery plan, inaccurate ultrasound findings, L-tube or intubation failure, and loss of data. Finally, 119 and 111 patients were assessed in the Ventilation and Apnea groups, respectively (Fig. 3).

There were no differences in patient characteristics between the groups (Table 1). For the primary outcome, gastric antral CSA changes on ultrasonography were greater in the ventilation group than the Apnea groups, but not significant (0.04 ± 0.3 and 0.02 ± 0.28, respectively, *p* = 0.225). Additionally, no significant differences were observed between the two groups in surgical grade and Levin tube suction (Table 2).

**Table 1:** Characteristics of patients undergoing pressure-controlled ventilation or non-ventilation during anesthesia induction

	Ventilation group (n = 119)	Apnea group (n = 111)	P-value
Age; y	50.8 (13.2)	50.7 (13.6)	0.901
Sex; female	53 (45%)	59 (53%)	0.235
Weight; Kg	66.3 (11.9)	65.1 (10.4)	0.515
Height; cm	165.5 (0.1)	164.0 (0.1)	0.215
BMI*	24.1 (3.0)	24.2 (2.7)	0.942
ASA physical status			
I II III	54 (45%) 60 (51%) 5 (4%)	48 (43%) 60 (54%) 3 (3%)	0.750
Underlying disease			
Diabetes mellitus Chronic kidney disease	15 (12.6%) 1 (0.8%)	17 (15.3%) 1 (0.9%)	0.573 0.367

Values are mean (SD) or number (proportion). *, body-mass index

**Table 2:** Gastric antral cross-sectional area, laparoscopic surgical grade and amount of gastric volume

	Ventilation group (n = 119)	Apnea group (n = 111)	P-value
Cross-sectional area; cm^2^			
Pre-induction (PI) Post-intubation (PT) Changes^*^	3.97 (1.36) 4.01 (1.45) 0.04 (0.3)	4.16 (1.32) 4.15 (1.41) 0.02 (0.28)	0.36 0.502 0.225
Laparoscopic surgical grade			
1 2 3 4	43 (36%) 68 (57%) 8 (7%) 0 (0)	52 (47%) 52 (47%) 7 (6%) 0 (0)	0.249
Gastric volume; mL			
Air Fluid Total	16.4 (17.6) 14.1 (18.0) 30.5 (28.6)	15.6 (13.5) 13.9 (16.7) 29.5 (25.8)	0.779 0.990 0.943

Values are mean (SD) or number (proportion). ^*^, (PT-PI)/PI

**Fig. 4:**
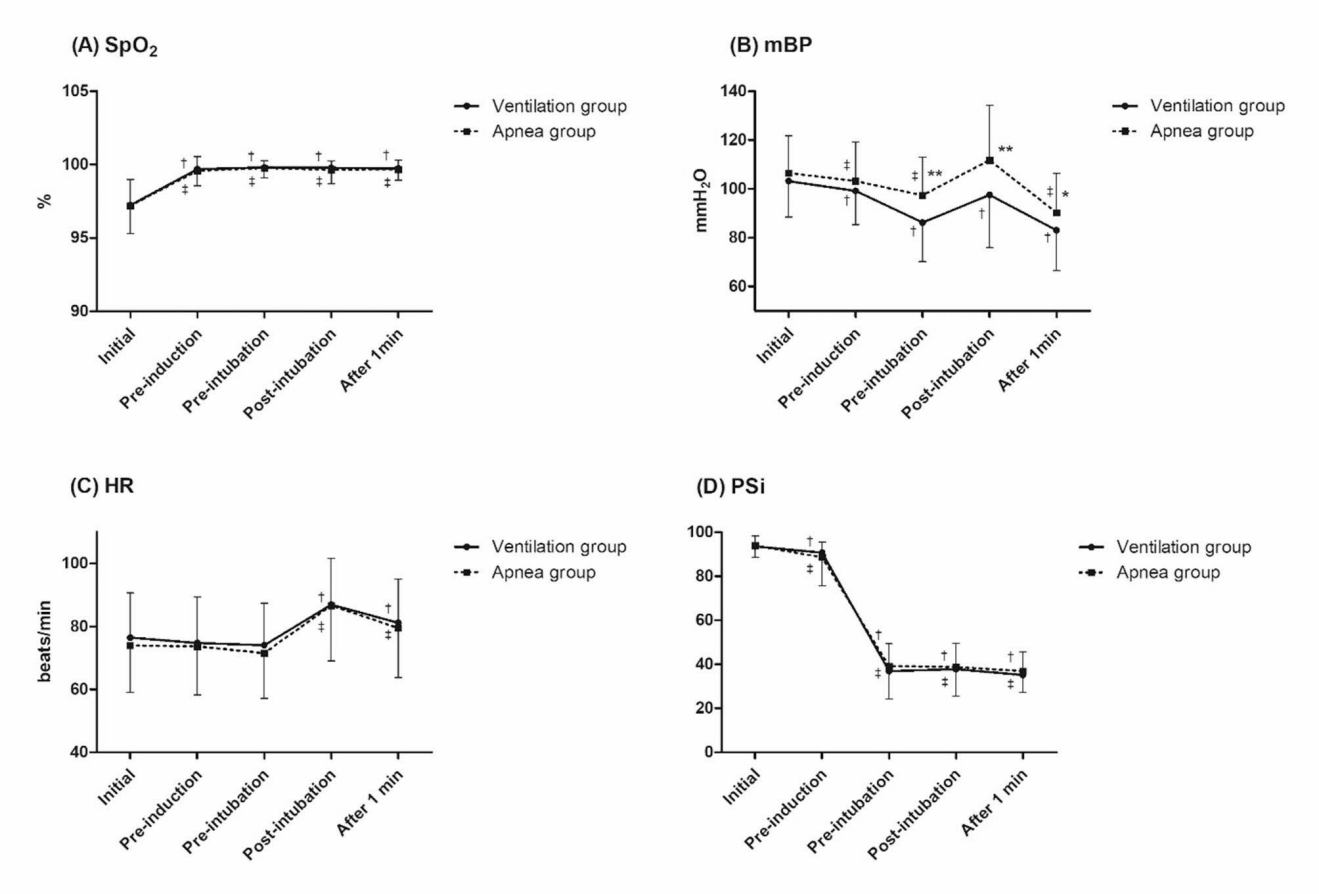
Vital sign changes during anesthesia induction. SpO_2_, saturation of percutaneous oxygen; mBP, mean blood pressure; HR, heart rate; PSi, patient state index. Data were analyzed using a repeated measures general linear model. * *p* < 0.05 compared with Ventilation group; ** *p* < 0.005 compared with Ventilation group; † *p* < 0.05 compared with ‘Initial’ in Ventilation group; ‡ *p* < 0.05 compared with ‘Initial’ in Apnea group

Changes in vital signs during induction were analyzed by repeated measures general linear model. Oxygen saturation was maintained at a safe level during induction in both groups. Mean blood pressure significantly changed in both groups over time (*p* < 0.001), and there was a significant difference in degree of change between the two groups (*p* = 0.006). This shows the stabilization of blood pressure via the effect of vasodilation of inhalational anesthetic gas in the ventilation group. Heart rate showed a similar trend in both groups, increasing about 10–15 times/min immediately after intubation, but rate increases were not dangerous and stabilized after one minute. The PSi decreased to the level of general anesthesia before intubation in a similar pattern in both groups (Table 3; Fig. 4).

**Table 3:** Values of various hemodynamic parameters

	Initial	Pre-induction	Pre-intubation	Post-intubation	After 1 min
SpO_2_ (%)					
V group	97.2 ± 1.7	99.7 ± 0.9	99.8 ± 0.5	99.8 ± 0.5	99.7 ± 0.5
A group	97.2 ± 1.9	99.6 ± 1.0	99.8 ± 0.7	99.6 ± 1.0	99.7 ± 0.7
P-value	0.820	0.333	0.667	0.156	0.452
MBP (mmHg)					
V group	103.1 ± 14.6	99.2 ± 13.8	86.2 ± 16.0	97.5 ± 21.6	83.0 ± 16.7
A group	106.4 ± 15.0	103.1 ± 16.2	97.3 ± 15.7	111.7 ± 22.5	90.2 ± 16.0
P-value	0.095	0.049	< 0.001	< 0.001	0.001
HR (beats/min)					
V group	76.4 ± 14.3	74.7 ± 14.7	74.0 ± 13.4	86.9 ± 14.7	81.1 ± 13.9
A group	74.0 ± 15.0	73.6 ± 15.4	71.5 ± 14.3	86.6 ± 17.6	79.5 ± 15.7
P-value	0.204	0.589	0.161	0.894	0.395
PSi					
V group	93.7 ± 4.7	90.8 ± 4.9	36.9 ± 12.4	37.8 ± 11.6	35.1 ± 10.3
A group	94.0 ± 5.3	89.7 ± 8.9	39.1 ± 14.8	38.7 ± 13.2	36.9 ± 9.8
P-value	0.614	0.251	0.221	0.581	0.191

Values are mean ± SD. SpO_2_: saturation of percutaneous oxygen, MBP: mean arterial blood pressure, HR: heart rate, PSi: patient state index, V group: ventilation group, A group: Apnea group

## Discussion

We hypothesized that gastric insufflation would increase even when mask ventilation was performed at an appropriate inspiratory pressure compared with no mask ventilation, but the difference in the two outcomes, the changes in gastric antral CSA and the laparoscopic grade, was not statistically significant. It can be inferred that an inspiratory pressure of 15 cmH_2_O is similarly safe to non-ventilation for avoiding excessive gastric insufflation. Additionally, the change in mean blood pressure was more stable in the Ventilation group compared with the Apnea group. This means that vital sign deterioration due to induction and intubation can be further stabilized by adequate delivery of inhalational anesthetic gas through mask ventilation.

During anesthesia induction, mask ventilation is required after loss of consciousness and before endotracheal intubation to provide adequate oxygenation to the patient. It is common practice for an anesthetist to apply a facemask to the patient’s face with one hand while squeezing an anesthetic circuit ventilation bag with the other hand. At this time, oxygen and anesthetic gas delivered to the facemask through the anesthetic circuit ventilation bag can enter the esophagus and the trachea through the oral cavity. When gas inflow into the esophagus increases, gastric distension occurs, which increases intragastric pressure, and pulmonary aspiration due to reflux of gastric contents [1–3]. Even if mask ventilation with a minimum pressure is performed, the air inflow into the stomach cannot be completely blocked [1, 4], and the amount is inevitably different because of the anatomical diversity of the patient’s airway. Eventually, excessive gastric insufflation can block the surgeon’s view during laparoscopic surgery, making the operation more difficult.

General anesthesia is known as a risk factor for gastric aspiration and the mortality rate within 30 days of aspiration pneumonia is close to 30% [15]. Therefore, it is important to prevent aspiration. Rapid sequence induction that minimizes the time until intubation after the patient’s respiratory reflex disappears (approximately one minute) is recommended when reflux of gastric contents is expected, such as insufficient fasting time or underlying disease [16]. Before anesthesia induction, sufficient preoxygenation is provided, and after administration of a high dose of neuromuscular blocker, endotracheal intubation is performed with shortening mask ventilation or with no mask ventilation [15]. The apnea method is used to reduce the risk of pulmonary aspiration caused by air inflow into the stomach due to mask ventilation. If preoxygenation is sufficiently performed, it takes more than two minutes for oxygen saturation to decrease from 100 to 95% after apnea initiation [17, 18]. Therefore, apnea for about one minute does not worsen oxygen saturation, so it can be applied to patients without hypoxia. Gentle mask ventilation is now considered acceptable during rapid sequence induction and intubation after loss of consciousness while reducing the hypoxia compared to apnea [1]. This study supports the previous papers that mask ventilation in the rapid sequence induction technique is safe by directly confirming gastric ultrasonography, surgeon’s view, and Levin tube suction. In addition, this study is significance in that it directly confirmed the degree of gastric insufflation through laparoscopy and proved that there was no correlation with gastric ultrasonography.

We predicted that gastric insufflation was more likely to occur if mask ventilation was performed compared with no mask ventilation during anesthesia induction, but our results did not support this prediction. Several reasons can be considered: First, this study was conducted by referring to the minimal inspiratory pressure that did not cause gastric insufflation during mask ventilation in previous studies. Thus, it is possible that significant gastric insufflation did not occur in the Ventilation group during anesthesia induction. Second, patients who were expected to have difficulty in mask ventilation were excluded from enrollment. Therefore, more research is needed on patients who may have difficulty with mask ventilation.

This study has some limitations. First, the dose and duration of rocuronium were different between the two groups. We wanted to reproduce the anesthesia situation as realistically as possible. For rapid sequence intubation, many anesthesiologists use rocuronium in an increased dose than usual, and shorten the time to intubation. Second, we did not measure tidal volume. In mask ventilation with an inspiratory pressure of 15 cmH_2_O, the tidal volume varied from patient to patient and for each breath. A more reliable result would have been possible if the patient’s tidal volume was recorded and analyzed. Third, real-time ultrasonography was not performed. Because we measured gastric antral CSA before and after induction, we could not confirm the actual change pattern during induction. Fourth, we performed this study in patients with no risk of gastroesophageal reflux or hypoxia who were within the normal body-mass index range. These results are not generalizable to broader populations, such as patients with comorbidities affecting gastroesophageal reflux, and patients with morbid obesity. Therefore, this study should be evaluated in various patient populations in future studies.

In conclusion, pressure-controlled mask ventilation of 15 cmH_2_O with the two-handed technique can support stable vital signs by maintaining oxygen supply and anesthesia depth, without increasing the risk of gastric insufflation, and without disturbing the surgical field. Furthermore, according to our results, the gentle mask ventilation method during rapid sequential induction appears acceptable.

## Data Availability

The datasets used and analyzed during the current study are available from the corresponding author on request.

## Abbreviations

ASA: American Society of Anesthesiologists
CO_2_: carbon dioxide
CSA: cross-sectional area
PSi: patient state index
